# Black Soldier Fly (*Hermetia illucens*) Larvae as a Protein Substitute in Adverse Food Reactions for Canine Dermatitis: Preliminary Results Among Patients

**DOI:** 10.3390/vetsci12010068

**Published:** 2025-01-17

**Authors:** Nuttawan Srifawattana, Yuthana Phimolsiripol, Pinpanit Boonchuay, Kannika Na-Lampang, Promporn Piboon, Sonthaya Umsumarng, Korakot Nganvongpanit

**Affiliations:** 1 Small Animal Hospital, Faculty of Veterinary Medicine, Chiang Mai University, Chiang Mai 50200, Thailand; nuttawan.p@cmu.ac.th; 2 Faculty of Agro-Industry, Chiang Mai University, Chiang Mai 50100, Thailand; yuthana.p@cmu.ac.th (Y.P.); pinpanit.b@cmu.ac.th (P.B.); 3 Faculty of Veterinary Medicine, Chiang Mai University, Chiang Mai 50100, Thailand; kannika.nalampang@cmu.ac.th (K.N.-L.); promporn.piboon@cmu.ac.th (P.P.); sonthaya.u@cmu.ac.th (S.U.)

**Keywords:** hypoallergenic diet, canine dermatitis, sustainable protein, food sensitivity, insect-based protein, dogs nutrition

## Abstract

Can black soldier fly (BSF) larvae be a protein substitute for managing adverse food reactions (AFRs) in dogs, specifically those with dermatitis? We evaluated BSF larvae’s safety, tolerance, and effectiveness as a novel protein source in dogs with AFRs. Sixteen dogs, including eight healthy controls and eight diagnosed with AFRs, were fed a diet containing BSF larvae for four weeks. Skin conditions, including dermatological lesions and Pruritus Visual Analog Scale (PVAS) scores, and gastrointestinal signs, including relevant clinical signs, stool consistency, and hematological and biochemical parameters, were monitored. No significant gastrointestinal side effects were observed. The control and AFRs groups maintained stable body weights, while stool consistency scores did not change significantly. Moreover, skin reaction data obtained before the study were collected through interviews with the owners. The mean PVAS scores in the AFRs group were significantly higher (*p* < 0.05) during the self-control period (6.0 ± 1.6) when compared with other periods. However, no significant differences (*p* > 0.05) were observed between the pre-study baseline (2.0 ± 1.1) and scores at 2 weeks (2.3 ± 1.5) and 4 weeks (2.4 ± 1.2) of feeding with BSF-based food, which indicated that BSF larvae did not exacerbate pruritic symptoms. Hematology and blood chemistry remained within normal ranges in all dogs, indicating no adverse effects on overall health. BSF larvae were well tolerated by AFR-affected and control dogs, with no adverse clinical outcomes. The findings suggest that BSF larvae may offer a better, sustainable alternative protein source for managing AFRs in dogs, particularly in those on hypoallergenic diets. Further long-term studies are needed to confirm these results and assess the broader implications of BSF larvae diets on canine health.

## Simple Summary

This preliminary study investigated the potential of black soldier fly (BSF) larvae as a protein substitute for managing adverse food reactions (AFRs) in dogs with canine dermatitis. Sixteen dogs, including eight healthy controls and eight with diagnosed AFRs, were fed a BSF larvae-based diet for four weeks. Researchers monitored skin conditions (dermatological lesions and Pruritus Visual Analog Scale scores), gastrointestinal signs, stool consistency, and hematological and biochemical parameters. Results showed no significant gastrointestinal side effects, and both groups maintained stable body weights with consistent stool quality. In the AFR group, pruritus scores during the self-control period were significantly higher, but BSF-based feeding did not exacerbate pruritic symptoms. Blood tests showed no adverse health effects, indicating that BSF larvae were well tolerated by both groups. These findings suggest BSF larvae could be a viable, sustainable protein alternative for hypoallergenic diets in dogs with AFRs, although further long-term studies are needed to validate these outcomes.

## 1. Introduction

The black soldier fly (BSF), with the scientific name *Hermetia illucens*, is commonly found in tropical and temperate climates located between 45 degrees north and 40 degrees south latitude. BSF is an insect that does not transmit diseases and is not considered a pest. The adult flies are black and can reach a length of up to 20 mm [1,2]. Previous studies have found that black soldier fly larvae contain 37–63% protein in their dry weight, 7–39% fat in their dry weight, and provide 2900 kcal/kg [3]. BSF larvae have long been used as food for animals [4,5] and are used as a protein source due to their high protein content. In particular, there have been extensive studies on their use in feeds for poultry, swine, and fish [3].

Adverse food reactions (AFRs) in dogs is a term used to describe any abnormal response to an ingredient in their diet [6]. It primarily involves the skin and gastrointestinal system and can be broadly classified into food allergies (immunologically mediated) and food intolerances (non-immunologically mediated) [7]. Adverse food reactions in dogs are estimated to account for about 1–2% of all canine diseases [8,9,10]. Although, among dogs with skin diseases specifically, AFRs are more prevalent, with rates ranging from 0 to 24%. This variation is due to breed predisposition, environmental influences, and diagnostic methods [11]; however, the prevalence of AFRs in dogs with dermatological signs was 12% (16 of 130) [10]. AFRs can occur at any age, though they often manifest in young to middle-aged dogs [8,9,10]. Certain breeds may be more predisposed to AFRs, including German Shepherds, West Highland White Terriers, Labrador Retrievers, Golden Retrievers, Cocker Spaniels, and Pugs [9,10,12]. The common clinical signs involve two major systems in the body: the dermatological and gastrointestinal systems [13,14]. The dermatological symptoms of AFR are most commonly characterized by itching (pruritus), which affects the ears, feet, and abdomen. These symptoms often present as recurrent skin infections, otitis externa, and atopic dermatitis [12]. The gastrointestinal symptoms include vomiting, diarrhea, flatulence, and bloating. Additionally, there have been reports of a large number of dogs and cats with AFRs showing symptoms of vomiting and/or diarrhea [15].

Dermatological signs predominated when the study was performed by dermatologists, wherein 261 dogs (71%) presented dermatological signs only, 10 dogs (3%) showed only gastrointestinal signs, and 97 dogs (27%) exhibited both gastrointestinal and dermatological signs [15]. Elimination diets have been considered the gold standard for diagnosing AFRs [7,16,17]. This process involves feeding the dog a novel protein or hydrolyzed protein diet for 8–12 weeks. If symptoms are resolved, the previous diet is gradually reintroduced to identify the offending allergen. This novel protein diet, or hydrolyzed protein diet, is then fed exclusively to the dog for at least 8 to 12 weeks [18,19] for an animal with dermatological clinical signs and 2–4 weeks [20,21] for an animal with gastrointestinal disease. The management of AFRs involves eliminating the offending food component from the diets of these dogs, often using hypoallergenic diets (novel protein or hydrolyzed protein diets) [6]. However, long-term dietary control is essential for preventing recurrence.

The prevalence of adverse food reactions, particularly in dogs with dermatological and gastrointestinal manifestations, is becoming more commonly recognized in the veterinary field. This is because most food allergies are triggered by proteins in the diets of dogs [14,17,18]. This study investigated the potential of BSF larvae as an alternative protein source for dogs diagnosed with AFRs. The experimental feed was formulated to replace conventional protein sources with BSF larvae while also incorporating essential additives required for preservation and large-scale pet food production. The primary objective was to evaluate the effectiveness and safety of this novel protein source in managing AFRs in dogs. This experimental food is designed to be commercially viable, incorporating essential additives needed for large-scale pet food production to ensure effective canning and preservation.

## 2. Materials and Methods

### 2.1. BSF Larvae Meal Preparation

Black soldier fly larvae (*Hermetia illucens*, BSFL) were reared using hemp by-products provided by Atlanta Medicare Co., Ltd., Chiang Mai, Thailand. Briefly, 6-day-old BSFL were fed dry hemp meal (mesh size < 80) as the main feed for 8 days. The 14-day-old BSFL was collected, cleaned with tap water, and frozen at −18 °C. Before use, the frozen BSFL was thawed and dried in a hot air oven (FD 115, Binder, Tuttlingen, Germany) at 70 °C for 16 h until the residual moisture content was 1.5–2.1% (*w*/*w*). The dried BSFL was then subjected to a size reduction process using a commercial blender (HR2118/02, Batam City, Indonesia) and used as an ingredient in the formulation of dog food. The proximate analysis, microbiological quality, amino acid profile, and fatty acid composition of BSFL meal were recorded as supplementary data (Tables S1–S4).

### 2.2. Formulation of BSFL Feed for Dogs

The dog food formula used in this study consisted of a commercially available adult dog food formula produced by Asian Alliance International Public Company Limited, Thailand, wherein the protein source was replaced with BSF larvae (Table 1).

### 2.3. Experimental Animals

This project was approved by the Animal Ethics Committee of the Faculty of Veterinary Medicine, Chiang Mai University (Approval No. R19/2567). Since this study was a clinical trial involving client-owned dogs, it referenced data from the Dermatology Clinic of the Small Animal Hospital, Faculty of Veterinary Medicine, Chiang Mai University, from the past four years. Approximately 20–30 dogs per year were diagnosed with canine atopic dermatitis (CAD) and adverse food reactions (AFRs). Accordingly, the study involved 16 client-owned dogs. This group included 8 healthy dogs and 8 dogs diagnosed by veterinarians with food-related adverse skin reactions (Table 2). The medical history information for each dog in the AFR group during the challenge diet protocol used to diagnose AFR was provided in Supplementary Data S1.

The diagnosis of AFR was made using standard procedures [7]. AFR dogs were managed at the dermatology clinic of a small animal hospital. All dogs were treated for ectoparasite control using isoxazoline products (e.g., sarolaner, Simparica^®^, a formulation containing hydrolyzed soy protein). Dogs were excluded if they were diagnosed with ectoparasite infestations or flea bite hypersensitivity (Table 3).

Dogs with bacterial pyoderma or Malassezia infections were treated until the infections resolved before they could begin the food elimination trial. AFR dogs were maintained on food elimination diets for 8–12 weeks, using commercial hydrolyzed diets throughout the diagnostic process. Dogs with uncontrolled or untreated infections, including ectoparasites (e.g., fleas, scabies), bacterial infections, dermatophytosis, or Malassezia, were excluded from the study until these conditions were resolved. Only after successful resolution of these issues were the dogs allowed to proceed with the food elimination trial (Table 4).

In cases where dogs did not respond to commercial hydrolyzed elimination diets, the protocol was adjusted to a home-cooked diet consisting of a single novel protein and a single novel carbohydrate source that the owner had not previously provided. However, dogs on home-cooked diets were excluded from this study due to time constraints and management limitations. If a dog failed to respond to the elimination diet, further diagnostic procedures were conducted to assess for canine atopic dermatitis (CAD) [7]. These dogs were not included in our study.

#### 2.3.1. Inclusion Criteria

a. The following dogs were diagnosed with AFRs at a dermatologic clinic in a small animal hospital for at least three months:

1. Dogs that had been treated with certified ectoparasite control products (e.g., isoxazoline, macrocyclic lactones, imidacloprid, fipronil, or S-methoprene) were included, while those who had exhibited flea bite hypersensitivity were ruled out.
2. Dogs that had undergone treatment for skin infections before starting a food elimination diet, if applicable.
3. Dogs classified under the pruritic skin syndrome diagnosis group.

b. The following healthy dogs without skin symptoms or itching were included in the control group:

1. Dogs that had undergone a preliminary health examination by a veterinarian.
2. Dogs that showed no signs of illness.
3. Dogs whose owners were able to manage and control their care and feeding regimen.

#### 2.3.2. Exclusion Criteria

1. Dogs with abnormal vital signs such as abnormal mucous membrane moisture, capillary refill time (CRT), rectal temperature, heart rate, heart sounds, pulse rate, respiratory rate, lung sounds, and hydration status.
2. Dogs with other systemic conditions such as liver or kidney disease.
3. Dogs receiving immunosuppressive drugs.

### 2.4. Study Design

The experimental design employed in this study was a multiple-time series design. The dogs were divided into two groups: a control group of 8 healthy dogs with no skin symptoms or itching and an AFR group of 8 dogs diagnosed with food-related adverse skin reactions, who had been on a food-elimination diet for at least 8–12 weeks. Both groups were fed a diet with BSF larvae as the protein source for four weeks to evaluate the acute effects of the diet over a shorter time period. All dogs were monitored for clinical outcomes and adverse reactions. These included itching and licking the body, groin, or abdomen. Adverse reactions among the studied dogs also included licking their paws, ear infections, and opportunistic bacterial skin infections.

### 2.5. Data Collection

The dogs were fed continuously for four weeks, and their skin conditions were monitored throughout the study period. Data on skin lesions, adverse reactions in the gastrointestinal tract, itching, licking the body, groin, abdomen, and/or paws, ear infections, and bacterial skin infections were collected. Moreover, data on skin reactions, before inclusion in the study, were recorded from interviews with the owners of the dogs. The data were divided into two categories: data collected by veterinarians and data collected by dog owners.

### 2.6. Veterinarian-Collected Data

The dogs were scheduled for vet visits before and after the 2- and 4-week feeding periods.

2.6.1. General data used for monitoring health status, including body weight, body temperature, respiratory rate, and heart rate, were collected.

2.6.2. Dermatological examinations, including skin cytology and lesion assessments, were recorded throughout the feeding period.

2.6.3. Hematology and blood chemistry were analyzed to assess overall health, including the following:

1. Complete Blood Count (CBC): hemoglobin (Hgb), hematocrit (Hct), red blood cell count (RBCS), red blood cell indices, white blood cell count (WBCs), differential count, absolute neutrophil count, and platelet count.
2. Blood Chemistry: creatinine, blood urea nitrogen (BUN), alanine transaminase (ALT), alkaline phosphatase (ALP), total protein, and albumin.

### 2.7. Owner-Collected Data

2.7.1. Food consumption behavior

2.7.2. Behaviors involving licking the body, groin, abdomen, or paws, along with dogs with ear infections and bacterial skin infections.

2.7.3. Stool consistency scores, based on Middelbos et al. [22], were rated on a 5-point scale as follows:

1 = hard, dry pellets; small hard mass;

2 = hard-formed, dry stool; stool remains firm but soft;

3 = soft, formed, and moist stool; retains shape;

4 = soft, unformed stool; assumes shape of the container;

5 = watery, liquid that can be poured.

2.7.4. Itching severity (Pruritus Visual Analog Scale (PVAS) scores) for owners, based on Hill et al. [23], rated on a 10-point scale as follows:

0 = Normal dog: Itching is not a problem for my dog.

2 = Very Mild Itching (Occasional Episodes): My dog is slightly itchier than I consider normal.

4 = Mild Itching (Semi-Frequent Episodes): My dog does not itch when eating, playing, exercising, or is distracted.

6 = Moderate Itching (Frequent Episodes): My dog itches periodically throughout the day but not when eating, playing, exercising, or being distracted. Itching occurs at night when observed.

8 = Severe Itching (Prolonged Episodes): Itching disrupts my dog’s sleeping, eating, and exercise behaviors. The itching continues even when my dog is distracted.

10 = Extreme Itching (Severe/Continuous Itching): My dog does not stop itching, regardless of surroundings or commands, and needs to be physically restrained from itching.

For the evaluation of PVAS scores, we included data from the medical records of each dog during the period prior to the food challenge protocol used for diagnosing AFR, which we defined as the self-control period. In the control group, PVAS scores were obtained from medical records covering the two months before their enrollment in the study.

### 2.8. Statistical Analysis

Data comparisons were made before and after the use of BSF food. The values were analyzed using R 4.41 and R-studio build764. Data were then analyzed using both descriptive and inferential methods. Median, quartile (Q)1, Q3, and interquartile range (IQR) were used to describe the data, and Wilcoxon signed rank was used to determine the difference within the group at each period. Each period was used as the independent variable, and PVAS scores were used as dependent variables, while the Wilcoxon rank-sum test was used to find the difference between groups during each period. Statistical significance was determined at *p* ≤ 0.05.

## 3. Results

**Figure 1:**
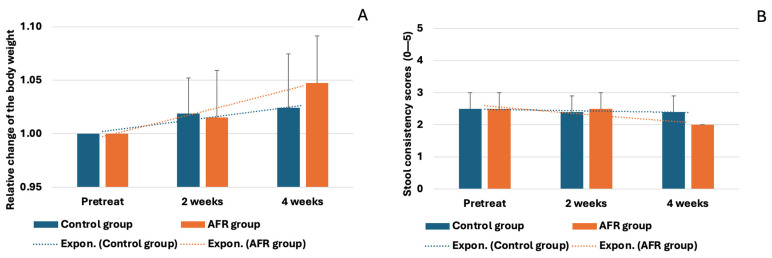
Relative change in body weight in the control and AFR groups (**A**); average (bar = SD) stool consistency scores in the control and AFR groups (**B**).

Throughout the 4-week study period, none of the dogs refused the food provided. All dogs were exclusively fed this formula, with no additional food sources. Furthermore, no gastrointestinal side effects were observed, such as vomiting or diarrhea. The body weights of the dogs in both groups (Figure 1A) showed no significant changes (*p* > 0.05) over the 4 weeks. Stool consistency 2/5 and 3/5 scores also remained stable, with no significant differences (*p* > 0.05) between baseline and study period measurements (Figure 1B).

**Figure 2:**
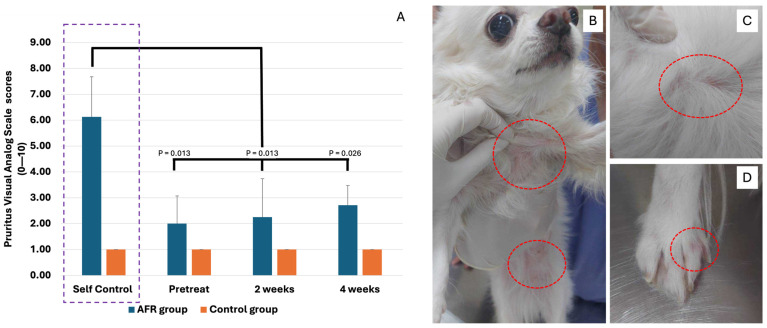
The Pruritus Visual Analog Scale (PVAS) scores for each dog in the control and AFR groups (**A**). No significant differences (*p* > 0.05) were observed between the pretreatment, 2-week, and 3-week periods in either group. However, in the AFR group, PVAS scores during these three time periods were significantly different (*p* < 0.05) when compared with the self-control period. Representative photos of skin lesions on the trunk (**B**), back (**C**), and feet (**D**) of this dog were excluded from this study in the second week.

All dogs in the control group maintained a PVAS score of 1 throughout the study (Figure 2). In the AFR group, four out of eight dogs initially had a PVAS score of 3, while the remaining dogs started with a score of 1. After two weeks on the BSF food, the scores between the two groups aligned, with only one dog experiencing an increase in PVAS score from 3 to 5. This dog was removed from the study after the second week (Figure 2). By the fourth week, three dogs in the AFR group exhibited a PVAS score increase from 1 to 3.

The overall median PVAS score for the control group remained at 1 with an IQR of 0. In the AFR group, the initial median score was 2 with an IQR of 2, which remained unchanged in the second week and shifted to a median of 3 with an IQR of 0 by the fourth week. In this group, there were no significant changes in PVAS scores from pretreatment to the second week (*p* = 1.000) or from the second to the fourth week (*p* = 0.447). Similarly, no significant change was observed when comparing PVAS scores from pretreatment to the fourth week (*p* = 0.447). During the self-control period of the AFR group, the median PVAS score was 6 with an IQR of 0.5. However, there were significant differences (*p* < 0.05) between the self-control and pretreatment PVAS scores (*p* = 0.013), as well as between self-control and both the second (*p* = 0.013) and fourth weeks (*p* = 0.026).

**Figure 3:**
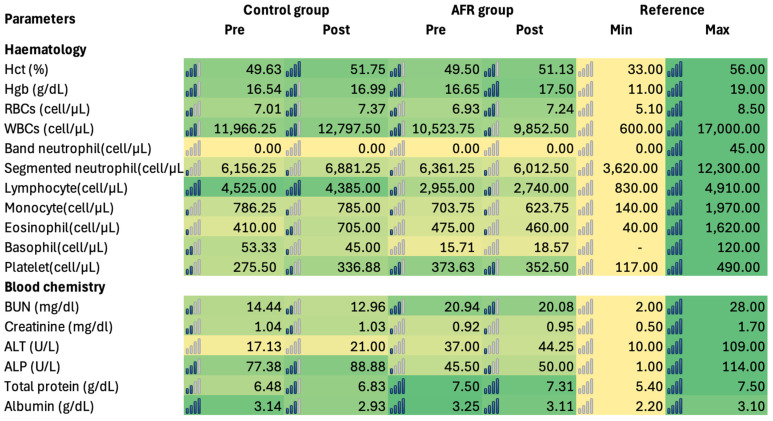
Average hematology and blood chemistry values for the control and AFR groups for pre- and post-feeding periods of BSF food (D).

Hematology and blood chemistry data (Table S5) before and after four weeks of BSF feed intake showed no significant differences (*p* > 0.05). None of the dogs exhibited values outside the standard range for key parameters, including Hct, WBCs, BUN, creatinine, ALT, and ALP (Figure 3).

## 4. Discussion

The relevance of this study lies in demonstrating the potential of using BSF-based food in dogs with AFRs. This would involve utilizing BSF larvae as a potentially promising alternative protein source in dogs displaying adverse food reactions (AFRs), particularly among those manifesting canine dermatitis. The results of this study indicate that BSF larvae-based food was well-tolerated by both healthy dogs and those with AFRs, with no significant (*p* < 0.05) gastrointestinal or dermatological side effects observed over the two-week feeding period. Notably, no dogs in either group experienced vomiting or diarrhea during this study, indicating that replacing the original protein source in the diet with BSF larvae protein did not negatively affect GI health under these conditions. However, further studies are needed to evaluate its suitability for dogs with more severe or varied gastrointestinal sensitivities. The findings contribute to the growing body of research supporting insect-based proteins in veterinary diets, particularly for dogs with dietary sensitivity.

Previous research, such as the study by Freel et al. [21], has shown that BSF larvae could serve as a viable source of protein for dogs, thereby supporting normal health outcomes without adverse reactions. The study by Freel et al. [21] evaluated the acceptance, safety, and digestibility of diets with varying levels of BSF larvae and oil. Twenty beagles were fed diets with up to 20% BSF larvae or 5% oil, all of which were well accepted. Another experiment with 56 dogs assessed digestibility using control and BSF diets. Health and nutritional metrics were monitored over 28 days. This study found no significant differences in body weight between the groups regarding normal stool characteristics, health scores, and blood chemistry values. These results indicate that using BSF larvae and oil at high levels did not adversely affect the dogs. Our study builds on these findings by demonstrating that BSF larvae can be a novel protein source in elimination diets for dogs with AFRs. However, unlike some hydrolyzed or extensively processed proteins, BSF larvae are a natural, minimally processed option, which may appeal to dog owners looking for sustainable and hypoallergenic dietary alternatives. The BSF food used in our study showed very high palatability because, during the 2-week study, no dog refused the food. Research by Jarett et al. [22] on the palatability and digestibility of insect proteins revealed that dogs readily accepted BSF-based diets, aligning with the high acceptance rate observed in the current study. These outcomes further validate that BSF larvae are a sustainable and nutritionally adequate protein source for dogs, including those with dietary sensitivities such as AFRs. Moreover, a study by Mancini et al. [24] explored the use of insect-based proteins for animal feed and suggested that BSF larvae could serve as a high-quality protein source that can replace traditional animal proteins in dog diets. This study confirmed that BSF larvae can be effectively utilized as an alternative source of protein without compromising health indicators. The results also echo the findings of Bosch et al. [25], which focused on the nutritional value and digestibility of BSF larvae in dog food, demonstrating that insect-based diets could support normal health parameters in dogs.

Concerning clinical outcomes, the PVAS scores of the AFR group revealed no statistically significant variations throughout the study. While 50% of the AFRs group started with PVAS scores of 3, most remained stable or improved slightly by the second week. One dog, whose itching symptoms worsened, was excluded from the study, which may have affected the overall results. Nevertheless, the lack of worsening pruritus in the AFR group during the early stages of the dietary change suggests that BSF larvae protein does not exacerbate skin conditions in most AFR cases. This is an encouraging result, particularly given the challenge of finding novel proteins that do not trigger allergic responses. In contrast to the AFRs group, the control group maintained consistently low PVAS scores throughout the course of the study, which further supports the safety of BSF larvae as a protein source in healthy dogs. Furthermore, hematological and blood chemistry parameters for both groups remained within normal ranges, suggesting an absence of adverse impacts on overall health. These findings are crucial, as some novel protein diets can lead to nutrient imbalances or suboptimal health outcomes over time. The stable body weights and stool consistency scores also indicate that BSF larvae provide adequate nutrition and gastrointestinal health, aligning with earlier studies on their digestibility and palatability [3].

Despite these positive findings, there are limitations to consider. First, the sample size of this study was relatively small, which may limit the generalizability of the results. Another limitation of our study was that the number of dogs was low due to the limited availability of confirmed AFR cases at our teaching hospital. Future studies should include larger populations of dogs with different breeds, ages, and levels of sensitivity to AFRs. Additionally, the short duration of the study (four weeks) may not have been long enough to observe more subtle or long-term effects of BSF larvae on canine skin health. Extending the feeding period in future studies would provide a more comprehensive understanding of the long-term safety and efficacy of the BSF larvae as a protein source. However, the positive trend observed with BSF food suggests that future, long-term studies should be designed to further validate its use in AFR dogs. Another concern is the inclusion of certain ingredients in the food formulation, which could have contributed to the skin reaction observed in one dog. As noted by Cianferoni and Spergel [26], as well as Gaschen and Merchant [27], food allergies are typically triggered by a protein, but other ingredients, such as corn, wheat, or soy, can also provoke reactions [28]. In food-allergic dogs, dogs with a tendency to allergies may have a more hypersensitive immune response than normal dogs. The possible immune mechanisms include hypersensitivity reactions of types I, III, and IV [29,30,31,32,33,34,35]. Importantly, 35–48% of food-allergic dogs are reported to be allergic to more than one type of food, while 10–15% have exhibited gastrointestinal symptoms as well [36,37]. Dogs in the food allergy group may have responded to the tested commercial food, which contained other components that may induce hypersensitivity. Future studies may consider testing with single protein sources.

## 5. Conclusions

This study highlights the potential of BSF larvae as a novel protein source for dogs experiencing AFRs, particularly those with skin-related symptoms. The absence of significant adverse effects, coupled with stable health markers and positive feedback from pet owners, suggests that BSF larvae could serve as a valuable component in hypoallergenic and elimination diets for dogs. However, further research with broader coverage of sample size and long-term duration would be necessary to confirm the efficacy of the BSF as a sustainable and safe food option for the canine population. The findings indicate that BSF larvae hold promise as a viable protein substitute in commercial dog food formulations, especially for dogs diagnosed with AFRs. Given the high prevalence of AFRs in dogs, which often present as dermatological or gastrointestinal symptoms, the inclusion of novel proteins, such as BSF larvae, provides a promising alternative for dietary management. The stability of hematological and biochemical parameters, and the lack of adverse clinical reactions, have confirmed the potential of BSF-based diets for dogs with food sensitivities. This offers a hypoallergenic alternative to traditional proteins such as chicken, beef, or soy. In addition, the sustainability of BSF production is a noteworthy advantage. BSF larvae are easy to farm and represent an environmentally friendly protein source, addressing growing concerns about the ecological footprint of conventional animal-based diets. The results of this study could pave the way for incorporating BSF larvae into hypoallergenic diets for dogs with AFRs, potentially reducing reliance on common proteins that may trigger allergic reactions. Additionally, during the food testing period, there were other uncontrolled factors, such as differing management strategies, care practices, and post-exercise management techniques, as well as environmental conditions and bathing, which could also complicate skin symptoms. In conclusion, this study supports the potential of BSF larvae as a safe, effective, and well-tolerated protein substitute for dogs with AFR, particularly in those exhibiting dermatological symptoms. Beyond offering a novel protein source, BSF larvae present a sustainable, eco-friendly alternative to conventional proteins. With further research, BSF-based diets have the potential to become a crucial strategy for managing food sensitivities in dogs while benefiting canine health and supporting environmental sustainability.

## Figures and Tables

**Table 1:** Ingredient list for dog feed.

Ingredient	Percentage (%)
Water	70.58
Insect meat protein	17.00
Rice flour	8.00
Modified tapioca starch	2.00
Tricalcium phosphate	0.50
Thickening agent (guar gum)	0.50
Potassium chloride	0.40
Magnesium sulfate	0.40
Vitamins and minerals (premix)	0.30
Choline chloride	0.20
Salt	0.10
Ferrous sulfate	0.01
Zinc	0.01
Copper chelates	0.002
Selenium	0.001

**Table 2:** Information on dogs included in the experiments.

Status	Sex	Age (Month)	Weight (kg)	Breed
Healthy (*n* = 8)	Female	5	13.50	Mongrel
	Female	108	3.20	Chihuahua
	Female	5	17.00	Mongrel
	Female	24	6.20	Chihuahua
	Female	72	17.30	Mongrel
	Male	180	11.00	Mongrel
	Male	5	15.00	Mongrel
	Male	24	21.00	Mongrel
AFRs (*n* = 8)	Female	108	3.40	Pomeranian
	Female	132	3.54	Chihuahua
	Female	96	2.25	Chihuahua
	Female	96	3.30	Chihuahua
	Female	96	3.00	Pomeranian
	Female	144	2.65	Chihuahua
	Female	36	23.10	American Pit Bull
	Male	120	3.25	Chihuahua

**Table 3:** Additional information collected during diagnosis of the disease in all eight dogs in the AFR groups.

Dogs	Ectoparasite Control	Infection (Cytology Check)			Set 2 Favrot’s Criteria [7]							
		Pyoderma	Dermatophytosis	Malassezia	1	2	3	4	5	6	7	Score
1	✓	×	×	×	✓	×	✓	✓	✓	✓	✓	6
2	✓	×	×	×	✓	✓	✓	✓	✓	✓	✓	7
3	✓	×	×	×	✓	✓	✓	✓	✓	✓	✓	7
4	✓	×	×	×	×	×	✓	✓	✓	✓	✓	5
5	✓	×	×	×	×	✓	✓	✓	×	✓	✓	5
6	✓	×	×	×	✓	×	✓	✓	×	✓	✓	5
7	✓	×	×	×	✓	✓	✓	✓	×	✓	✓	6
8	✓	×	×	×	✓	✓	✓	✓	✓	✓	✓	7

**Table 4:** Monitoring of clinical signs in all dogs of the AFR group during food elimination diets but before being challenged by BSF protein in this study.

Criteria [7]	Dogs															
	1		2		3		4		5		6		7		8	
	Pre.	Post.	Pre.	Post.	Pre.	Post.	Pre.	Post.	Pre.	Post.	Pre.	Post.	Pre.	Post.	Pre.	Post.
PVAS score	6	3	8	3	6	3	6	1	5	1	5	1	6	3	6	1
Pododermatitis	1	0	1	0	1	1	1	0	1	0	1	0	1	0	1	0
Axillary and inguinal area	1	0	1	0	1	0	1	0	0	0	0	0	1	0	1	0
Face (periocular and mouth)	1	0	1	0	1	0	1	0	1	0	0	0	1	0	1	0
Otitis	0	0	0	0	0	0	1	0	0	0	0	0	0	0	0	0
Stool score	3	3	2	2	2	2	2	2	5	3	3	3	2	2	3	3

Pre. = pre diagnosis of adverse food reactions, Post. = post diagnosis of adverse food reactions.

## Data Availability

The data presented in this study are available within the article’s figures and tables.

## Supplementary Materials

The following supporting information can be downloaded at https://www.mdpi.com/article/10.3390/vetsci12010068/s1, Table S1: Proximate analysis of black soldier fly larvae (BSFL); Table S2: Microbiological quality of black soldier fly larvae (BSFL); Table S3: Amino acid profile of black soldier fly larvae (BSFL); Table S4: Fatty acid composition of black soldier fly larvae (BSFL); Table S5: Hematology and blood chemistry data of the dogs; Table S6: Clinical score of the dogs; Supplementary Data S1: The medical history information for each dog in the AFR group.
