# Supplementary material for: Black Soldier Fly (Hermetia illucens) Larvae as a Protein Substitute in Adverse Food Reactions for Canine Dermatitis: Preliminary Results Among Patients
Source: Vet Sci. 2025 Jan 17;12(1):68. doi: 10.3390/vetsci12010068 (PMC11768785; doi:10.3390/vetsci12010068)
Supplement: Supplementary file 1 [file vetsci-12-00068-s001.zip › Supplementary Data S1.pdf]

Supplementary data 1

The food elimination diet, food rechallenge, and food challenge results for all eight dogs in the Allergic Reaction Food (ARF) group were recorded before being included in the study involving the food challenge with Black Soldier Fly (BSF).

Subject No. 1

A 9-year-old female Pomeranian was treated at the Dermatology Clinic of the Small Animal Hospital, Faculty of Veterinary Medicine, Chiang Mai University. She had been under monitoring and care for the past three years, since the age of six, due to pruritic skin disease. The dog was kept indoors, with consistent ectoparasite control and regular skin health management.

Before starting a food elimination diet, the dog had a Pruritus Visual Analog Scale (PVAS) score of 6. Chronic pododermatitis and erythema were observed in the axillary and inguinal areas, as well as around the mouth and periocular regions. No issues with the excretory system were reported.

After initiating a food elimination trial using a hydrolyzed diet, the dog showed significant improvement within 10 weeks of follow-up without requiring any medication. After maintaining control with the hydrolyzed diet for 15 weeks, the owner introduced treats as part of a dietary challenge. This led to a marked increase in the PVAS score, with the dog exhibiting excessive paw licking. The treats were suspected to contain chicken, liver, or other ingredients (snacks). Following the discontinuation of the treats, the dog's condition improved, with a decrease in the PVAS score and no new skin lesions observed.

Table : Monitoring of clinical signs in Subject No. 1 from the AFR group during food elimination diets and food challenge phases

Criteria	Pre-diagnosis of adverse food reactions	Food elimination diet control	Food Challenge
PVAS score	6	3	6
Pododermatitis	1	0	1
Axillary and inguinal area	1	0	0
Face (Periocular and mouth)	1	0	0
Otitis	0	0	0
Stool Score	3	3	3

Subject No. 2

An 11-year-old female Chihuahua was receiving treatment at the Dermatology Clinic of the Small Animal Hospital, Faculty of Veterinary Medicine, Chiang Mai University. The dog alternated between living indoors and outdoors, and her owner provided a varied diet consisting of both commercial pet food and human food.

Between the ages of 5 and 7, the dog was closely monitored and treated for recurrent pyoderma, which was followed by the development of pruritic skin disease that had been present since early childhood. A food trial was initiated after the dog's symptoms significantly improved through dietary control using a hydrolyzed diet for 12 weeks. However, when the owner reverted to the dog's original diet—composed of commercial pet food containing corn, poultry meal, rice, full-fat soybean, chicken oil, brewer's dried yeast, liver digest, lecithin, fish oil, iodized salt, vitamins and minerals, antioxidants, and food coloring—the dog developed symptoms of itching, foot licking, and inflammation, with redness observed on the abdomen, face, and inguinal areas.

Following a diagnosis of an adverse food reaction, the dog was placed on a food elimination diet using hydrolyzed food. Since then, the dog's condition has been successfully managed. The owner reported that the dog occasionally exhibited mild itching, but it was minimal. The Pruritus Visual Analog Scale (PVAS) score was recorded as 3, with no visible lesions. The dog had remained free of recurrent skin problems for the past three years under this dietary regimen.

Table : Monitoring of clinical signs in Subject No. 2 from the AFR group during food elimination diets and food challenge phases.

**Criteria**	**Pre-diagnosis of adverse food reactions**	**Food elimination diet control**	**Food Challenge**
PVAS score	8	3	5
Pododermatitis	1	0	1
Axillary and inguinal area	1	0	1
Face (Periocular and mouth)	1	0	1
Otitis	0	0	0
Stool Score	2	2	2

Subject No. 3

An 8-year-old Chihuahua was presented for diagnosis at the Dermatology Clinic of the Small Animal Hospital, Faculty of Veterinary Medicine, Chiang Mai University. The dog was kept indoors and fed a home-cooked diet consisting of chicken, pork bone broth, cabbage, rice, bread, cheese, and unspecified treats.

For more than several years prior to presentation, the dog had been exhibiting symptoms including foot licking, pododermatitis, redness around the eyes and mouth, and pruritus. A review of the dog's medical history revealed a history of at least three episodes of otitis externa over the years before its admission to the Dermatology Clinic of the Small Animal Hospital, Faculty of Veterinary Medicine, Chiang Mai University.

An elimination diet trial using hydrolyzed food was conducted over 12 weeks. During this period, the owner reported a 50% reduction in pruritus, with only mild pododermatitis, characterized by redness without itching. Following this, a dietary rechallenge was performed using the original home-cooked diet, which included rice mixed with chicken and pork bone broth.

Within the first week of the rechallenge, the dog's symptoms began to recur, with marked redness and itching becoming evident by the second week. The owner reverted to the hydrolyzed diet and continued it consistently for the recommended 12-week period before undergoing further evaluations.

Table : Monitoring of clinical signs in Subject No. 3 from the AFR group during food elimination diets and food challenge phases.

**Criteria**	**Pre-diagnosis of adverse food reactions**	**Food elimination diet control**	**Food Challenge**
PVAS score	6	3	6
Pododermatitis	1	1	1
Axillary and inguinal area	1	0	0
Face (Periocular and mouth)	1	0	1
Otitis	0	0	0
Stool Score	2	2	2

Subject No. 4

A 3-year-old female American Pit Bull was presented with severe deep pyoderma, external otitis, and severe pododermatitis. She was treated at the Dermatology Clinic of the Small Animal Hospital, Faculty of Veterinary Medicine, Chiang Mai University, where infection control measures were implemented. Culture results identified *Staphylococcus pseudintermedius* as the causative organism. Treatment was continued for three months, during which cytology confirmed the resolution of the infection. Despite this, the dog continued to exhibit symptoms of external otitis, pododermatitis, and persistent foot licking.

The owner reported that the foot-licking behavior had begun before the infection and that the dog had not responded well to steroid treatments in the past. The dog lived in a garden environment and was fed a diet consisting of raw food (including chicken and pork) along with free access to commercial dog food.

A food elimination trial with a hydrolyzed diet was initiated, leading to significant improvement in pruritus, foot licking, pododermatitis, and otitis over a 14-week period. Subsequently, at the owner's request, a step-by-step approach to food reintroduction was undertaken. Each trial tested one source of protein and one source of carbohydrate. Increased a Pruritus Visual Analog Scale (PVAS) score and the development of skin lesions were observed when the dog was challenged with pork, chicken, and soy. In contrast, challenges with beef, buffalo meat, fish, rice, and quinoa did not trigger itching or skin lesions, as detailed in the table.

To prepare for additional food trials, the dog was returned to the hydrolyzed diet to ensure a stable condition for 10 weeks before testing new food options.

Table : Monitoring of clinical signs in Subject No. 4 from the AFR group during food elimination diets and food challenge phases.

**Criteria**	**Pre-diagnosis of adverse food reactions**	**Food elimination diet control**	**Food Challenge**
PVAS score	6	1	6
Pododermatitis	1	0	1
Axillary and inguinal area	1	0	1
Face (Periocular and mouth)	1	0	1
Otitis	1	0	1
Stool Score	2	2	2

Subject No. 5

A 7-year-old spayed female Pomeranian was presented with pruritus, red rashes on the face, and frequent ear scratching. The dog had a history of recurrent surface pyoderma, occurring 3–4 times per year. Hair loss was observed on the distal limbs. Upon further inquiry, the owner reported that the dog consistently experienced soft stools, which they had attributed to a canned food diet (composed of chicken meat, modified starch, chicken liver, water, sugar, wheat gluten, gum, salt, and vitamin E) and therefore had not considered it problematic. The dog lived indoors with several other small-breed dogs, none of whom displayed any abnormalities.

Following initial treatment, a food elimination trial using a hydrolyzed protein canned diet was implemented. Over the course of 13 weeks, the dog showed significant improvement, including firmer stools, the resolution of alopecia, and complete regrowth of fur. There was no evidence of scratching or pruritus during this period.

As part of a dietary challenge, the original canned food was reintroduced. Within the first three days, the dog developed diarrhea and mild redness of the face, periocular areas, and pinnae. However, upon resumption of the hydrolyzed diet, all symptoms resolved without additional treatment within 5–7 days. The owner chose to continue feeding the hydrolyzed diet to maintain the dog's improved condition.

Table : Monitoring of clinical signs in Subject No. 5 from the AFR group during food elimination diets and food challenge phases.

**Criteria**	**Pre-diagnosis of adverse food reactions**	**Food elimination diet control**	**Food Challenge**
PVAS score	5	1	1
Pododermatitis	1	0	0
Axillary and inguinal area	0	0	0
Face (Periocular and mouth)	1	0	1
Otitis	0	0	0
Stool Score	5	3	5

Subject No. 6

An 8-year-old Chihuahua was brought in for treatment of persistent foot licking. The owner reported observing this behavior for over three years, with inflammation and pododermatitis developing in the year leading up to its visit to the Dermatology Clinic at the Small Animal Hospital, Faculty of Veterinary Medicine, Chiang Mai University.

According to the dog's history, previous treatments with antipruritic medications, including steroids and oclacitinib acid, had been ineffective. The dog was kept indoors and fed a breed-specific commercial diet composed of rice, maize, dehydrated poultry proteins, wheat gluten, animal fats, hydrolyzed animal proteins, beet pulp, minerals, vegetable fibers, fish oil, soya oil, fructo-oligosaccharides, borage oil, marigold meal, glucosamine from fermentation, hydrolyzed cartilage, preservatives, and antioxidants.

A food elimination trial using a hydrolyzed diet was initiated, which resulted in significant improvement in the dog's Pruritus Visual Analog Scale (PVAS) scores and resolution of pododermatitis lesions on the feet within 10 weeks. Complete resolution of the lesions was observed by the 14-week follow-up.

Subsequently, a dietary rechallenge was performed using the breed-specific commercial diet. Within two weeks, the dog began exhibiting itching and licking in the inguinal and axillary areas. The rechallenge was discontinued, and the hydrolyzed diet was resumed. The owner continued the hydrolyzed diet for long-term management.

Table: Monitoring of clinical signs in Subject No. 6 from the AFR group during food elimination diets and food challenge phases.

**Criteria**	**Pre-diagnosis of adverse food reactions**	**Food elimination diet control**	**Food Challenge**
PVAS score	5	1	3
Pododermatitis	1	0	0
Axillary and inguinal area	0	0	1
Face (Periocular and mouth)	0	0	0
Otitis	0	0	0
Stool Score	3	3	3

Subject No. 7

A 12-year-old female Chihuahua was referred from the Cardiology Clinic at the Small Animal Hospital, Faculty of Veterinary Medicine, Chiang Mai University, for consultation regarding pruritus and dermatological concerns. The dog presented with redness, lichenification, and hyperpigmentation affecting the periocular area, face, both ears, axillary and inguinal regions, as well as the interdigital areas with pododermatitis. The dog was primarily kept indoors, with occasional outdoor exposure, and was fed a variety of foods, including pork, fish, chicken, sausage, bread, rice, and table scraps. The owner reported that these symptoms had been ongoing for over 4–5 years without any previous treatment. However, due to the dog's concurrent heart condition, the owner sought veterinary care at the Small Animal Hospital, including a consultation at the Dermatology Clinic.

A food elimination trial was conducted using hydrolyzed commercial diets. Follow-up evaluations every four weeks revealed significant improvement. By weeks 8 and 12, the owner reported a 50% reduction in licking and itching. Additionally, dermatological lesions showed gradual healing, with no noticeable signs of inflammation by weeks 12 to 16. The owner expressed satisfaction with the overall results.

A dietary rechallenge was conducted using processed sausage containing pork, beef, or poultry, along with flavor enhancers, preservatives, and binders. This led to a recurrence of symptoms. Within two weeks of the rechallenge, the dog began licking and itching all over its body, accompanied by redness on the feet, as well as the axillary and inguinal regions.

Table : Monitoring of clinical signs in Subject No. 7 from the AFR group during food elimination diets and food challenge phases.

**Criteria**	**Pre-diagnosis of adverse food reactions**	**Food elimination diet control**	**Food Challenge**
PVAS score	6	3	5
Pododermatitis	1	0	1
Axillary and inguinal area	1	0	1
Face (Periocular and mouth)	1	0	0
Otitis	0	0	0
Stool Score	2	2	2

Subject No. 8

A 10-year-old male Chihuahua was presented at the Dermatology Clinic of the Small Animal Hospital, Faculty of Veterinary Medicine, Chiang Mai University, with symptoms of itching and foot licking (pododermatitis). The dog also exhibited redness and hair loss around the periocular and mouth areas, along with erythema on the pinnae. The dog was kept indoors, and the owner had several other dogs, none of which showed any abnormal symptoms. The dog had been displaying symptoms since a young age, and over the past 2–3 years, the owner had attempted to change its diet multiple times, trying various commercial dog foods made from meats such as fish, chicken, duck, and beef, all marketed as suitable and of high quality for dogs. Despite these dietary changes, the clinical signs persisted. At the time of presentation, the dog was being fed a plant-based diet but continued to show the same clinical symptoms.

A food elimination trial with a hydrolyzed diet was initiated, resulting in significant improvement. The itching nearly resolved within 10 weeks, and the dog continued on this diet for a total of 12 weeks. Following this, the owner introduced a plant-based protein challenge, which led to a noticeable increase in the Pruritus Visual Analog Scale (PVAS) score during the first week, as shown in the table. Based on these findings, it was decided to return to the hydrolyzed diet and maintain it consistently for optimal results.

Table: Monitoring of clinical signs in Subject No. 8 from the AFR group during food elimination diets and food challenge phases.

**Criteria**	**Pre-diagnosis of adverse food reactions**	**Food elimination diet control**	**Food Challenge**
PVAS score	6	1	3
Pododermatitis	1	0	0
Axillary and inguinal area	1	0	0
Face (Periocular and mouth)	1	0	0
Otitis	0	0	0
Stool Score	3	3	3
