# Supplementary material for: Black Soldier Fly (Hermetia illucens) Larvae as a Protein Substitute in Adverse Food Reactions for Canine Dermatitis: Preliminary Results Among Patients
Source: Vet Sci. 2025 Jan 17;12(1):68. doi: 10.3390/vetsci12010068 (PMC11768785; doi:10.3390/vetsci12010068)
Supplement: Supplementary file 1 [file vetsci-12-00068-s001.zip › Table S1.pdf]

Table S1 Proximate analysis of black soldier fly larvae (BSFL).

Composition	Content	Method
Ash (g/100 g)	12.4	AOAC 920.153 (2019)
Calories (Include dietary fiber, kcals/100 g)	430	Method of analysis for Nutrition Labeling (1993)
Carbohydrate (Include dietary fiber, g/100 g)	17.6	Method of analysis for Nutrition Labeling (1993)
Dietary Fiber (Total, g/100 g)	8.33	In-house method based on AOAC 985.29 (2019)
Fat (g/100 g)	17.2	In-house method based on AOAC 2008.06 (2019)
Moisture (g/100 g)	1.68	In-house method based on AOAC 925.09 (2019)
Protein (N×6.25, g/100 g)	51.1	In-house method based on AOAC 981.10 (2019)
