# Supplementary material for: Black Soldier Fly (Hermetia illucens) Larvae as a Protein Substitute in Adverse Food Reactions for Canine Dermatitis: Preliminary Results Among Patients
Source: Vet Sci. 2025 Jan 17;12(1):68. doi: 10.3390/vetsci12010068 (PMC11768785; doi:10.3390/vetsci12010068)
Supplement: Supplementary file 1 [file vetsci-12-00068-s001.zip › Table S2 Microbiological quality of black soldier fly larvae.pdf]

Table S2 Microbiological quality of black soldier fly larvae (BSFL).

Microbiological test	Content	Method
Aerobic plate count (CFU/g)	8,100	FDA BAM online, Chapter 3 (2001)
Campylobacter coli (in 25 g)	ND*	FDA BAM online, Chapter 7 (2001)
Campylobacter jejuni (in 25 g)	ND*	FDA BAM online, Chapter 7 (2001)
Clostridium perfringens (in 0.2 g)	ND*	FDA BAM online, Chapter 16 (2001)
Coliforms (Org/g)	<10	FDA BAM online, Chapter 4 (2020)
Enterococci spp. (CFU/g)	1,000	COMP, Chapter 10 (2015)
Escherichia coli (Org/g)	ND*	FDA BAM online, Chapter 4 (2020)
Listeria monocytogenes (in 25 g)	ND*	ISO 11290-1 (2017)
Salmonella spp. (in 25 g)	ND*	ISO 6579-1 (2017)/Amd.1 (2020)
Staphylococcus aureus (in 0.1 g)	ND*	FDA BAM online, Chapter 12 (2016)
Yeast and mold (CFU/g)	63	FDA BAM online, Chapter 18 (2001)

Note:

* ND = Not detected.
