# Supplementary material for: Black Soldier Fly (Hermetia illucens) Larvae as a Protein Substitute in Adverse Food Reactions for Canine Dermatitis: Preliminary Results Among Patients
Source: Vet Sci. 2025 Jan 17;12(1):68. doi: 10.3390/vetsci12010068 (PMC11768785; doi:10.3390/vetsci12010068)
Supplement: Supplementary file 1 [file vetsci-12-00068-s001.zip › Table S3 Amino acid profile of black soldier fly larvae.pdf]

Table S3 Amino acid profile of black soldier fly larvae (BSFL).

**Amino acids**	**Content (mg/100 g)**
Aspartic acid	4,930
Cystine	63.8
Glutamic acid	5,780
Glycine	1,930
Histidine	1,550
Hydroxylysine	11.8
Hydroxyproline	69.6
Isoleucine	2,080
L-Alanine	4,000
L-Arginine	3,380
Leucine	4,910
Lysine	2,870
Methionine	2,170
Phenylalanine	2,440
Proline	4,370
Serine	2,010
Threonine	1,970
Tryptophan	361
Tyrosine	3,100
Valine	3,140

Note:

- Limit of detection (LOD) and limit of quantitation (LOQ) are 5.0 and 10.0 mg/100 g, respectively.
- Amino acid profile was analyzed based on AOAC 994.12 (2019).
