# Supplementary material for: Black Soldier Fly (Hermetia illucens) Larvae as a Protein Substitute in Adverse Food Reactions for Canine Dermatitis: Preliminary Results Among Patients
Source: Vet Sci. 2025 Jan 17;12(1):68. doi: 10.3390/vetsci12010068 (PMC11768785; doi:10.3390/vetsci12010068)
Supplement: Supplementary file 1 [file vetsci-12-00068-s001.zip › Table S4 Fatty acid composition of black soldier fly larvae.pdf]

Table S4 Fatty acid composition of black soldier fly larvae (BSFL).

Fatty acids	Content (g/100 g)
Alpha-linolenic acid (C18:3, ALA, Omega-3)	0.53
Arachidic acid (C20:0)	0.04
Arachidonic acid (C20:4, Omega-6)	ND*
Behenic acid (C22:0)	0.02
Butyric acid (C4:0)	ND*
Capric acid (C10:0)	0.17
Caproic acid (C6:0)	ND*
Caprylic acid (C8:0)	ND*
Docosadienoic acid (C22:2, Omega-6)	ND*
Docosahexaenoic acid (C22:6, DHA, Omega-3)	ND*
Eicosadienoic acid (C20:2, Omega-6)	ND*
Eicosapentaenoic acid (C20:5, EPA, Omega-3)	ND*
Eicosatrienoic acid (C20:3, Omega-6)	ND*
Eicosenoic acid (C20:1, Omega-9)	0.01
Erucic acid (C22:1, Omega-9)	ND*
g-Eicosatrienoic acid (C20:3, Omega-3)	ND*
g-Linolenic acid (C18:3, Omega-6)	ND*
Heneicosanoic acid (C21:0)	0.05
Lauric acid (C12:0)	3.68
Lignoceric acid (C24:0)	ND*
Linoleic acid (C18:2, Omega-6)	4.75
Margaric acid (C17:0)	0.07
Margaroleic acid (C17:1)	ND*
Monounsaturated fat	3.33
Myristic acid (C14:0)	0.65
Nervonic acid (C24:1, Omega-9)	ND*

Fatty acids	Content (g/100 g)
Oleic acid (C18:1, Omega-9)	2.73
Omega-3	0.53
Omega-6	4.75
Omega-9	2.74
Palmitic acid (C16:0)	2.43
Palmitoleic acid (C16:1)	0.59
Pentadecanoic acid (C15:0)	0.07
Pentadecenoic acid (C15:1)	ND*
Polyunsaturated fat	5.29
Saturated fat	7.68
Stearic acid (C18:0)	0.48
Tetradecenenoic acid (C14:1)	0.01
Trans fat	0.06
Trans 9,12-Linolelaidic acid (C18:2trans)	ND*
Trans-Elaidic acid (C18:1trans)	0.06
Tricosanoic acid (C23:0)	ND*
Tridecanoic acid (C13:0)	0.01
Unsaturated fat	8.62

Note:

* ND = Not detected.

- Limit of detection (LOD) and limit of quantitation (LOQ) are 0.008 and 0.01 g/100 g, respectively.
- Fatty acid composition was analyzed by using in-house method STM No.03-010 based on AOAC 996.06 (2019).
